# Rat Riddance

**Published:** 1873-02

**Authors:** 


### RAT RIDDANCE.

A single dead rat may poison the atmosphere of a whole house for weeks and months and produce serious disease; hence it is unwise to use poison to get rid of them. They can be driven from a building in various ways, not involving the keeping of a dog or cat.

